# Effect of Seedling Nitrogen Condition on Subsequent Vegetative Growth Stages and Its Relationship to the Expression of Nitrogen Transporter Genes in Rice

**DOI:** 10.3390/plants9070861

**Published:** 2020-07-07

**Authors:** Hue Thi Nong, Ryota Tateishi, Chetphilin Suriyasak, Takuya Kobayashi, Yui Oyama, Wun Jin Chen, Ryo Matsumoto, Norimitsu Hamaoka, Mari Iwaya-Inoue, Yushi Ishibashi

**Affiliations:** Graduate school of Bioresource and Bioenviromental Sciences, Kyushu University, Mootoka 774, Fukuoka 819–0395, Japan; nthue86sh@gmail.com (H.T.N.); 2BE16007K@s.kyushu-u.ac.jp (R.T.); naiizzure@gmail.com (C.S.); 2BE17044N@s.kyushu-u.ac.jp (T.K.); oyama.yui.503@s.kyushu-u.ac.jp (Y.O.); 2BE18034K@s.kyushu-u.ac.jp (W.J.C.); matsumoto.ryo.700@s.kyushu-u.ac.jp (R.M.); nohamaoka@agr.kyushu-u.ac.jp (N.H.); mariino@agr.kyushu-u.ac.jp (M.I.-I.)

**Keywords:** N deficiency, N transporter genes, seedling stage, rice

## Abstract

Nitrogen (N) deficiency is one of the most common problems in soils, limiting crop growth and production. However, the effects of N limitation in seedlings on vegetative growth remain poorly understood. Here, we show that N limitation in rice seedlings restricted vegetative growth but not yield. Aboveground parts were affected mainly during the period of tillering, but belowground parts were sensitive throughout vegetative growth, especially during panicle development. At the tillering stage, N-limited plants had a significantly lower N content in shoots, but not in roots. On the other hand, N content in roots during the panicle development stage was significantly lower in N-limited plants. This distinct response was driven by significant changes in expression of N transporter genes during growth. Under N limitation, N translocation from roots to shoots was greatly sped up by systemic expression of N transporter genes to obtain balanced growth. N limitation during the seedling stage did not reduce any yield components. We conclude that the N condition during the seedling stage affects physiological responses such as N translocation through the expression of N transporter genes.

## 1. Introduction

Nitrogen (N) fertilizers are essential for crop growth and productivity [1]. In rice cultivation, the benefits of an appropriate split N topdressing are well documented and include mitigated N loss, improved N use efficiency [2]; improved photosynthesis efficiency, enhanced matter production capacity [3]; and increased number of spikelets per unit area and, ultimately, increased yield [3,4,5]. Insufficient N application at any growth stage leads to negative effects on growth and development, thus, affecting yield [6,7,8]. In the early growth stage, an inappropriate supply of N causes a failure to achieve vigorous seedling establishment [6]. Moreover, inadequate N supply at either the tillering and/or heading stage results in slow growth, less productive tillers, poor grain filling and reduced yield [8,9,10]. In fact, N deficiency is being repeatedly experienced by all plants [11]. The physiological effects of N deficiency have been intensively studied in many crops, along with the underlying molecular basis [12,13]. However, little is known about the possible impact of N limitation experienced by seedling plants on subsequent vegetative growth, and in turn yield in rice.

NO_3_^−^ and NH_4_^+^ are the two main inorganic N forms in soils; NH_4_^+^ is the main N source and the preferential form for rice in flooded environments [14,15]. N uptake by rice roots is mediated by nitrate transporters (NRTs) and ammonium transporters (AMTs). In rice, there are two families of NO_3_^−^ transporters (NRTs); these transport nitrate, nitrite, amino acids, peptides, phytohormones, and glucosinolates [16,17]. More than 80 NRT1/PTR (NITRATE TRANSPORTER 1/ PEPTIDE TRANSPORTER) and 4 NRT2 transporters, but only a few members, have been characterized [18,19,20]. *OsNRT2.1*, *OsNRT2.2*, and *OsNRT2.3a* are transcriptionally upregulated by NO_3_^−^ supply and need to interact with OsNAR2.1, which is an interacting partner protein of OsNRT2s required for nitrate uptake, while *OsNRT2.3b* and *OsNRT2.4* can function independently without OsNAR2.1 [18,19,20]. Knockout of *OsNRT2.4* did not affect rice growth or N uptake under conditions without N or with only NH_4_^+^ supply [21]. NH_4_^+^ transporters are responsible for membrane transport of NH_4_^+^ in plants. Rice expresses at least 10 putative AMTs in 4 groups; however, only limited information about expression patterns, localization, and transport activity is available under field conditions [22,23]. *OsAMT1.1* contributes significantly to NH_4_^+^ uptake under both low and high NH_4_^+^. A loss-of-function *OsAMT1.1* mutant significantly decreased total N transport from roots to shoots and repressed rice growth under low NH_4_^+^ [24]. *OsAMT1.2* was induced by NH_4_^+^ exclusively in roots, while *OsAMT1.3* was induced by N deficiency in roots [22]. *OsAMT2.1* was constitutively expressed in both roots and shoots irrespective of N supply, whereas *OsAMT3.1* expression was relatively weak [23].

In plants, stress memory refers to the ability of plants to remember past incidents and to use it for adapting to new challenges [25]. Accumulating evidence shows that pre-exposure to stress such as drought [26], heat [27], and osmotic stress [28] lead to alteration of the plant’s subsequent stress response, normally by producing a faster and/or stronger reaction as an adaptive strategy. For example, a short duration of drought caused Arabidopsis plants to wilt under the dehydration stress and then quickly recover after rehydration during a second drought stress, implying existence of stress memory, which improved their resistance to drought to achieve optimal growth [26]. However, the effect of stress memory in the agriculture field is a gap. As N deficiency occurs repeatedly, it raises the possibility that N stress memory experienced by seedling plants may also affect subsequent responses at a later vegetative stage. A comprehensive analysis of the changes in physiological and molecular N uptake and transport in rice plants that experience N limitation during the seedling stage would be partially helpful to understand this knowledge gap.

Thus, the objectives of this study were to examine (1) the effect of the N condition at the seedling stage on N accumulation and vegetative growth and (2) its relationship to the expression of N transporter genes.

## 2. Results

### 2.1. Analysis of Rice Growth at Different Growth Stages

**Figure 1:**
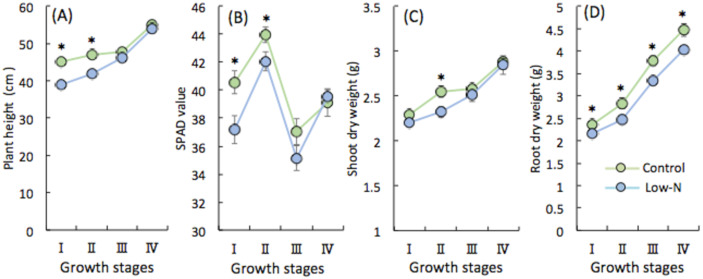
(**A**) Plant height; (**B**) SPAD value; (**C**) shoot dry weight; (**D**) root dry weight. I, II, III, and IV refer to before and after topdressing at tiller initiation and early panicle formation stages. Each point represents the mean value, error bars indicate mean ± SD of 5 replicates. * Significant difference (*p* < 0.05, Student’s *t*-test).

NH_4_^+^ is a primary N source and is preferentially taken up by paddy rice under the anaerobic conditions in flooded soil [9]. Here, we used NH_4_^+^ as a single N source to address the influence of the N condition during seedling development on the following growth stages in rice. We analyzed results at four dates: immediately before and 6 days after N topdressing at both tillering (stages I, II) and heading (stages III, IV). The SPAD value (leaf green color), plant height, and root dry weight were significantly lower in low-N plants (1/3 N of the control) than in the control plants during tillering (I, II), but shoot dry weight was generally not significantly different (Figure 1). Above ground, control plants seemed to be more vigorous during tillering, and low-N plants showed typical deficiency symptoms—restricted shoot growth (Figure 1A), pale green leaves with lower SPAD values (Figure 1B), and less dry biomass production (Figure 1C). However, these parameters at heading stage (III, IV) were not affected by low-N treatment of seedlings (Figure 1A–C). Therefore, we expected more complex root growth in low-N plants at tillering (I, II). Instead, root systems were compromised, with a significant decrease of biomass in low-N plants at all growth stages (Figure 1D). Thus, shoots and roots responded differently to N availability to seedlings during vegetative growth.

### 2.2. Accumulation of N in Shoots and Roots at Various Growth Stages

**Figure 2:**
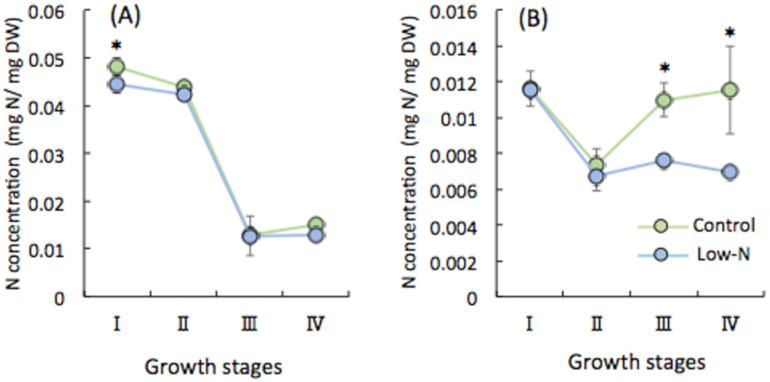
N contents in (**A**) leaves and (**B**) roots. I, II, III, IV refer to before and after topdressing at tiller initiation and early panicle formation stages. Each point represents the mean value, error bars indicate mean ± SD of 5 replicates. * Significant difference (*p* < 0.05, Student’s *t*-test).

To reveal the responses of shoots and roots during vegetative growth to seedling N condition, we analyzed the N contents at stages I, II, III, and IV. Leaves had a significantly lower concentration of N in low-N plants than in the control at tiller initiation (I) (Figure 2A), which indicates a physiological effect of N limitation at the seedling stage. However, there was no distinguishable difference at the later stages (III, IV). Moreover, there was a sharp decrease in N content from tillering (I, II) to panicle development (III, IV). Roots, in contrast, accumulated more N in both control and low-N plants during panicle development (III, IV) (Figure 2B). There were no differences in N accumulation between control and low N plants during tiller development (I, II), but N accumulation was significantly higher in control roots than in low-N roots at early panicle formation and heading (III, IV). These results provide strong evidence that rice growth during the vegetative stage is affected by the N environment experienced by seedlings. The reduced N input during the seedling stage affected aboveground organs more during tiller development (I, II), but belowground parts more later (III, IV). To explore the molecular mechanism of this process, we examined the transcript levels of N transporter genes in leaves and roots at each growth stage.

### 2.3. Gene Expression of N Transporter Genes

**Figure 3:**
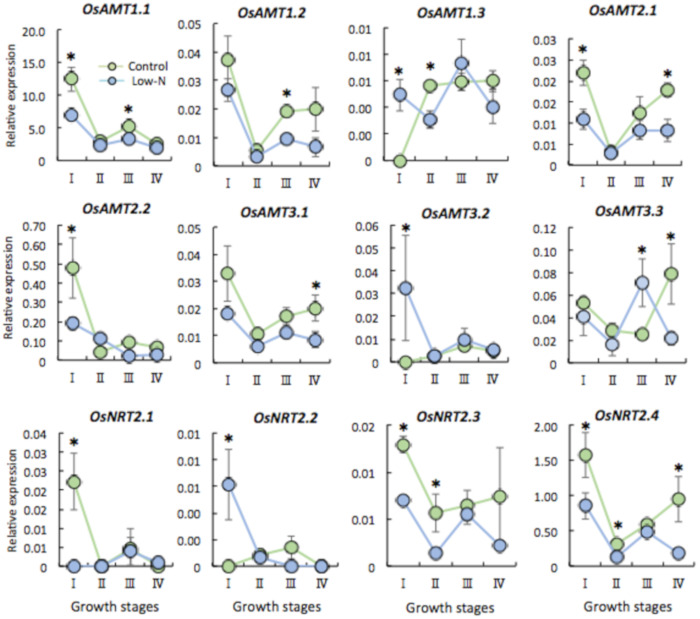
Relative expression of N transporter genes (NH_4_^+^ transporter genes, AMT; NO_3_^−^ transporter genes, NRTs) in leaves. I, II, III, IV refer to before and after topdressing at tiller initiation and early panicle formation stages. Each point represents the mean value, error bars indicate mean ± SD of 5 replicates. * Significant difference (*p* < 0.05, Student’s *t*-test).

We measured the expression of genes for ammonium transporters (*OsAMT1.1*, *1.2*, *1.3*; *OsAMT2.1*, *2.2*, *2.3*; and *OsAMT3.1*, *3.2*, *3.3*) and nitrate transporters (*OsNRT2.1*, *2.2*, *2.3*, *2.4*) in leaves and roots during vegetative growth. In leaves, the expression of the *AMT* genes changed significantly during the growth stages. Notably, most *AMTs* had low transcript levels in low-N plants throughout the growth stages (Figure 3). Many *AMTs* can be induced by NH_4_^+^ and repressed by N starvation [22,23,24]; however, the expected induction of *AMTs* by the supply of NH_4_^+^ in the first topdressing did not occur at the tiller development stage (II), and expression instead reached a minimum in both control and low-N plants. These genes were upregulated during panicle development (III, IV) (Figure 3), but expression was still low in low-N plants. The expression of NRT gene transcripts was similar, being low in low-N plants during tillering (I, II), indicating a correlation between gene expression and N content in leaves during tiller development. The expression of N transporters in later stages (III, IV) was low in low-N leaves, and therefore we expected the N content to be low, but leaf N content was equivalent to that in control leaves (Figure 2A). Thus, the promotion of N uptake in roots might be the cause of this phenomenon.

**Figure 4:**
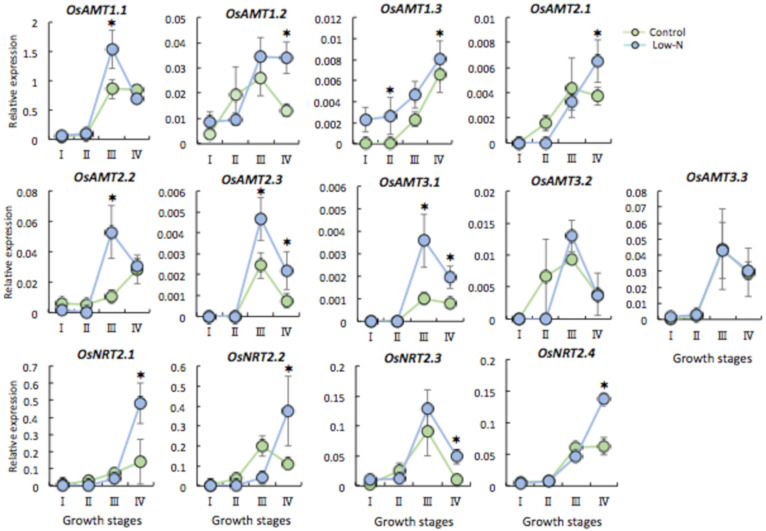
Relative expression of N transporter genes (NH_4_^+^ transporter genes, AMT; NO_3_^−^ transporter genes, NRTs) in root. I, II, III, and IV refer to before and after topdressing at tiller initiation and early panicle formation stages. Each point represents the mean value, error bars indicate mean ± SD of 5 replicates. * Significant difference (*p* < 0.05, Student’s *t*-test).

The expression of N transporter genes in roots also changed greatly during the growth stages (Figure 4). Interestingly, there was almost no difference in the levels of AMT transcripts between control and low-N plants during tiller development (I, II), unlike in shoots. During panicle development (III, IV), many *AMT* and *NRT* genes had higher expression levels in low-N plant roots than in the control (Figure 4). Thus, these results imply that N limitation in seedlings promotes N uptake by upregulation of N transporter genes in roots and its rapid translocation to shoots to achieve an optimal nutrient balance and promote growth later.

## 3. Discussion

N deficiency restricts plant growth and crop production. Many attempts have been made to analyze the expression of N transporter genes in response to N deficiency in young seedlings in rice [18,22,23,24,29], maize [30,31], and millet [32]. Here, we analyzed the effect of seedling N condition on the expression of N transporter genes in relation to subsequent vegetative growth in rice.

It is well documented that N availability determines many aspects of morphology, physiology, gene expression, and metabolites in rice [33]. We found that low N caused growth retardation during tiller development (I, II) but no difference later (III, IV) (Figure 1A–D). One explanation is that standard N topdressing of both control and low-N plants at the tiller initiation (I) and early panicle formation stages (III) could enhance later growth as a result of compensation and/or N stress memory effect. This rapid growth, achieving a similar plant height and aboveground dry biomass production in rice plants after N limitation during the seedling period, might partially compensate for the loss of nutrients under stress. These results showing the robust response of low N plants also implicate the existence of nutrient deficiency memory in agricultural fields, which has a similar effect as other abiotic stresses such as drought and heat [26,27]. In contrast to aboveground parts, root biomass production was decreased in low-N plants throughout subsequent growth (Figure 1D), so compensation did not work effectively in roots. It is noted that N deficiency affected dynamic growth of both shoots and roots. In the early vegetative stage, N deficit increases the root:shoot biomass ratio, presumably due to sustained investment in roots for N foraging to achieve balance with that of the control plant (data not shown). Thus, growth rates of Low-N plant roots is expected to be relative to shoot growth. At a later stage, the significantly low production of root biomass of low N plants over the time and the marked increase of the root:shoot ratio (stage II to III) implies that shoot growth was preferential by contrast to root growth of low N plants to obtain similar whole plant growth. Yet, these results raised the question of why the root dry weight was decreased, when plants are seen to develop a more extensive root system as a typical morphological response to low N in the short term [33,34]. To answer this question, we predicted that low N triggers a strategy of adaptation toward N starvation in both shoots and roots, manifested as the prompt remobilization of stored N and its transport via N transporter genes from roots to shoots. This hypothesis was confirmed by the analysis of the relationship between N content and the expression of *AMTs* and *NRTs* in both shoots and roots (Figure 3 and Figure 4). Plants respond to nutrient deficiency by inducing or repressing different sets of genes at specific times [35]. *AMT* and *NRT* genes were upregulated in roots and downregulated in shoots of low-N plants (Figure 3 and Figure 4), in contrast to N content in each organ. This shows that N was indeed transported from roots to shoots later. The upregulation of *AMT1.2*, *AMT1.3*, and *NRT2* (high affinity nitrate transporter) genes in low-N plant roots at stages III and IV (Figure 4) indicates that N uptake was enhanced by up-scaling transporter expression. Therefore, we expected N accumulation to be higher in low-N plant roots owing to the induction of transporter genes, but instead it was significantly lower. In shoots, downregulation of those genes (Figure 3) and a similar N accumulation between low-N and control plants (Figure 2A) at the later stage support the conclusion that low-N conditions promoted not only N uptake but also prompt translocation from roots to shoots. These results reveal that a systemic response may allow low-N plants to optimize N allocation so as to achieve optimal shoot growth in a strategy for adaptation to N limitation. Moreover, significant changes in the expression of *AMT* and *NRT* genes during the development of tillers (I, II) and panicles (III, IV) suggest that they have different roles at each stage. For example, the expression of *OsAMT1.3* and *OsNRT2.1* is induced by N deficiency [22,23,29], but we found that these genes showed different expression patterns at tiller initiation (I), which is most likely influenced by the effect of N limitation during the seedling stage. Moreover, their expression was marked, particularly during panicle development (III, IV), in the roots of plants that received normal N (Figure 4), implying that low-N treatment during the seedling period has long-lasting effects at the morphological, physiological, and molecular levels. Based on these results, the systemic but not local effect of N limitation experienced by seedling stage at root level was exhibited. In contrast to rice, NH_4_^+^-fed Arabidopsis plants failed to express systemic responses to localized N limitation in terms of stimulation of N uptake and lateral root growth, suggesting a local signal in regulation of NH_4_^+^ acquisition [36]. A split root experiment would be ideal to reveal a positive feedback whereby the promoted response at the vegetative stage induced by seedling N deficiency would lead to an increase in plant N uptake mediated by a systemic signal in rice.

Another explanation of the continued retardation of root growth in low-N plants even after N application (I, III) is the involvement of hormone accumulation, especially auxins. It is well documented that auxin plays a crucial role in initiation, emergence and development of lateral roots (LRs) mediated by auxin transporters that facilitate the distribution of indrole-3-acetic acid (IAA) between sink and source tissues [37,38]. We reasoned that the smaller root system might be due in part to a reduction in auxin accumulation, resulting in the inhibition of LR formation and thus low dry matter production. Differential distribution of auxin caused by N treatment in roots of two different NO_3_^−^ responsive-rice cultivars showed the stimulation of LR growth by greater auxin accumulation was enhanced in response to NO_3_^−^, not NH_4_^+^, and localized treatment with NH_4_^+^ decreased IAA level, with the consequent failure to stimulate LR growth in rice [39]. Moreover, our result is consistent with a result in millet grown under limitation. The result showed a small root system with a significant decrease in total root length, number of crown roots and LRs and density, and significantly lower auxin concentration in both shoots and roots [40]. Thus, in our study, N limitation during the seedling stage may generate a systemic repression of lateral root growth during vegetative growth, resulting in low production of dry biomass. However, other underlying mechanisms are also possible. Further effort is needed to explore how growth and development are regulated.

Besides the significant changes in N transporter gene expression in low N rice roots, we also noticed these changes in the control plant roots that received normal N supply across growth stages (Figure 4). Arguments that could be taken into account include distinct expression of the root zone-specific localization of the N transporter, especially AMTs, which may affect N uptake via N transport pathways and N allocation to shoots. It is accepted that apart from N availability, the expression of nitrogen transporter genes is tightly linked to tissue and development patterns [30]. In rice roots, the epidermis-expressed *OsAMT1.1* and *OsAMT 1.3* and the endodermis-expressed *OsAMT1.2* control uptake of NH_4_^+^ via the symplastic and apoplastic transport pathway, respectively [22]. Furthermore, cell type-specific localization of NRTs is associated with their expression and function in N signaling [40,41]. The tonoplast-localized *OsNRT1.1A* displays an NH_4_^+^-induced expression pattern and serves as an intracellular platform to facilitate the nuclear localization of NLP transcription factors, which play a central role in activating the expression of N utilization related genes [41]. While plasma membrane-localized *OsNRT1.1B* shows a NO_3_^−^ induced expression pattern, which enhanced NO_3_^−^ uptake and root-to-shoot transport and upregulated expression of NO_3_^−^-responsive genes, contributing to nitrate-use divergence between rice subspecies [40]. Thus, the distinct cell type-specific expression of individual N transporters in roots may enable them to express their different functions associated with N uptake and transport along growth stages. NH_4_^+^ fertilizer is quickly absorbed by root and rice roots are exposed to partial NO_3_^−^ nutrition by nitrification in the rice rhizosphere, resulting in the expression of NRTs. In addition, to respond to external N availability and the internal plant N status, N transporter gene expression is tightly controlled in roots by multiple regulatory mechanisms [22,23,42]. For instance, NH_4_^+^ and NO_3_^−^ regulate NH_4_^+^ uptake activity of *AtAMT1.3* via phosphorylation [42] or phosphorylation of some N transporter genes (*AMT1.1* and *NRT1.1* by CIPK23) to modulate their activity in response to the N condition, or epigenetic regulation through histone methylation to repress expression of the high-N-induced *AtNRT2.1* [13,43,44]. In our study, given the effect of N stress memory, it is a high possibility that epigenetic regulation such as DNA methylation and/or histone modification may be involved in regulation of expression of N transporter genes for N absorption and translocation.

It has long been known that nitrogen deficiency results in an accumulation of carbohydrates in leaves and roots, and modifies the allometric response of shoots and roots [45,46]. The different responses of shoots and roots to N status during the seedling stage suggests that low N plants exhibit a bias toward aboveground allocation, because root growth is more severely suppressed than shoot growth in all growth stages. Before topdressing, it is commonly assumed that low N plants put more energy into root growth for N foraging [45,46]. After topdressing, the allometric relationship of shoot and root growth seem unaffected by changes in N supply during the tillering stage (stage I, II) (Figure 1). However, it is likely that faster development of shoots of low N plants indicate more energy allocation toward the shoot to obtain balanced growth during the panicle development stage.

**Figure 5:**
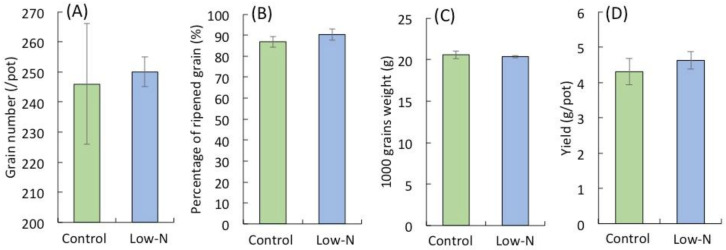
Yield components (**A**), Grain number per pot; (**B**), percentage of ripened grain; (**C**), 1000 grains weight) and yield (**D**). Control and low-N refer plants receive sufficient N and 1/3 that of the control during seedling stage, respectively. Error bars represent mean ± SD of 5 replicates.

Good seedling establishment is important for productivity and profitability of rice [47]. Since N accumulation in aboveground parts was comparable between low-N and control plants during panicle development (Figure 1 and Figure 2A) and N uptake during the vegetative growth stage contributes to the reproductive and grain filling stages via N translocation [48], we speculated that this explains the similarity in yield. There were no significant differences in yield and the yield components (percentage of ripened grains, grain number, and 1000-grain weight) between the treatments (Figure 5), suggesting that low N during the seedling period affects vegetative growth but not yield. Studies of the effects of N application during rice seedling establishment on grain yield show that high or stable yield can be achieved with less N input at the seedling stage, in particular in direct-sown rice [49,50,51,52]. Since direct sowing offers promise in place of transplanting in single rice cropping owing to its lower N requirement [49], this result will be helpful for rice production strategies.

In several crop species, the source–sink relationship is influenced by many factors such as nutrients, irrigation, light, defoliation [53,54,55]. Basically, roots are the source of inorganic N and are sinks for carbon (C), while leaves are often the major sink organs for N and sources of C [53]. Normally, sink–source balance is required for optimal growth and development of plants. In our study, by removing tillers to leave only the main stem, an artificial detillering system with less nutrient demand and a shortage of carbon might be created, and the sink–source relation may also be altered, possibly affecting the plant’s response to N. In response to this alteration, reduced whole-plant photosynthesis on plant function and decreased allocation of carbon to roots was often seen [56]. Moreover, removal of shoot biomass means N sink tissues are reduced, which may upregulate N storage in other parts of the plant such as in culm, which may play a role in plant recovery after grazing [57]. Root respiration and nutrient acquisition may also be reduced following defoliation [58]. Therefore, it is believed that a new adaptive sink–source balance, and probably a concomitant defect in N sink–source transition, was reestablished after cutting for ensuring steady-state growth. In response to nutrient limitation, it was also reported that respiration, nutrient absorption and allocation of C to roots were maintained or even increased, following removal/defoliation [59]. However, in our study these responses disappeared instead of a more severe level of reduction since significantly defective root biomass was exhibited in low N plants at all growth stages. The alteration of plant functioning due to the massive removal of aboveground biomass should also be one reason for this reduction in roots as described above. In addition, we do not rule out the possibility that a small additional N supply could be sufficient to support biomass to obtain a similar growth between low N and control plants at later stages since less demand of nutrients may be created.

Our study thus concluded that seedling N condition affects N translocation through the expression of N transporter genes at later vegetative stages as an adaptive strategy. This result can be extrapolated if this behavior is transmitted to the next generations. As an acquired adaptive trait exists, which in turn, is of potential benefit for practical agricultural systems.

## 4. Materials and Methods

### 4.1. Plant Material and Growth Conditions

Seeds of rice (*Oryza sativa* L.) ‘Nipponbare’ were germinated in the dark at 27 °C, sown in sterile sand in a glasshouse, and watered with distilled water. We planted three 15-day-old seedlings in 1/5000-a Wagner pots and grew them under field conditions at Kyushu University (33°67′ N, 130°42′ E). To minimize the inconsistency in mobilizing assimilates and nutrients among tillers and the possibility of indirect effects of the sink–source balance caused by differences in the number of tillers, all fully developed tillers were removed as they appeared to leave only the main stems. By removing tillers to leave only the main stem, an artificial detillering system with less nutrient demand might be created and the sink–source relation may also be altered, affecting plant growth rate. However, it was also reported that respiration, nutrient absorption and allocation of C to roots were maintained or even increased, following removal/defoliation of plant grown on nutrients [59]. Moreover, plants have evolved sophisticated mechanisms in response to changing environmental conditions; therefore, it is believed that a new adaptive sink–source balance was reestablished after cutting for ensuring their growth.

### 4.2. N Treatment

We used ammonium sulfate ((NH_4_)_2_SO_4_; 21% N) as the N source, which was applied 3 times during the rice growth stage as top-dressing. The rate in the low-N treatment was 1/3 that of the control during seedling stage (Figure S1). The following amounts were applied: 6.48 g/l (control) and 2.16 g/l (Low N) on the day of sowing (1 ml each), respectively; transplanting, 0.4 (control) and 0.133 (Low-N) g N/pot; and at the tiller initiation and early panicle formation stages, 0.4 g N/pot. We also applied P fertilizer (17.5% P) at 1.14 g/pot and K fertilizer (60% K) at 0.33 g/pot at transplanting. Stages I to IV refer to before and after topdressing at the tiller initiation and early panicle formation stages: (I) before, (II) 6 days after tiller initiation topdressing, (III) before, (IV) 6 days after early panicle formation topdressing.

### 4.3. SPAD Value, Length and Dry Weight of Shoots and Roots

All agronomic traits were measured in 3 plants per pot with 5 replicates. The SPAD value is the mean reading of the 3 uppermost fully expanded leaves per pot measured with a SPAD-502 meter (Konica-Minolta, Osaka, Japan). Plant height was the average of the 3 plants. Shoot and root samples were oven-dried at 80 °C to a constant weight and weighed for the calculation of dry biomass.

### 4.4. N Content

Dry leaf samples (uppermost fully expanded leaves) and root samples were ground into a fine powder, 50 mg of which was used for determining N content by the Kjeldahl method, then digested in H_2_SO_4_−H_2_O_2_ [41] for absorption spectrophotometry (U-1800 UV-Vis Spectrophotometer, Hitachi, Japan).

### 4.5. RNA Extraction and Quantitative Real-Time PCR

Total RNA from leaves and roots at growth stages I to IV was isolated by the SDS/phenol/LiCl method. cDNA was synthesized with ReverTra Ace reverse transcriptase according to the manufacturer’s instructions (Toyobo, Osaka, Japan). Quantitative real-time PCR was performed in a CFX Connect Real-Time PCR System (Bio-Rad, Hercules, CA, USA) with Thunderbird SYBR qPCR mix (Toyobo, Osaka, Japan) with initial denaturation at 94 °C for 2 min, followed by 40 cycles of denaturation at 94 °C for 20 s, annealing at a primer-specific temperature for 20 s, and extension at 72 °C for 20 s. The primer sequences are listed in Table S1. *OsActin* was used for normalization.

## Supplementary Materials

The following is available online at https://www.mdpi.com/2223-7747/9/7/861/s1: Table S1 Primer sequences used for quantitative real-time PCR.
