# Supplementary material for: Effect of Seedling Nitrogen Condition on Subsequent Vegetative Growth Stages and Its Relationship to the Expression of Nitrogen Transporter Genes in Rice
Source: Plants (Basel). 2020 Jul 7;9(7):861. doi: 10.3390/plants9070861 (PMC7412562; doi:10.3390/plants9070861)
Supplement: Supplementary file 1 [file plants-09-00861-s001.pdf]

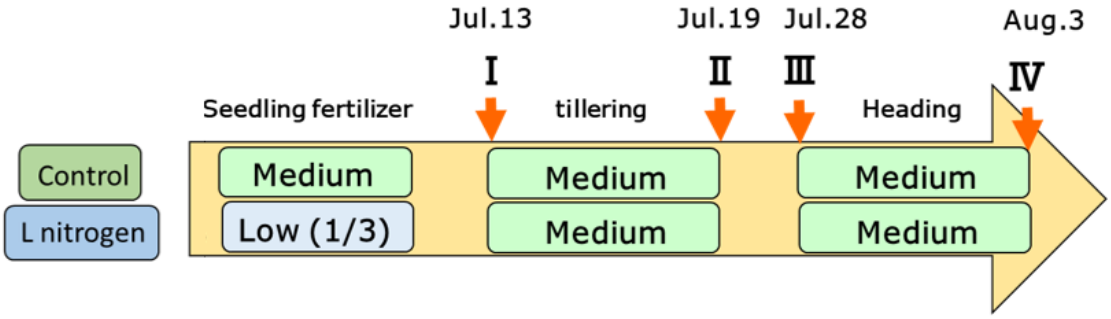

Figure S1. Experiment design of nitrogen supply at seedling stage. I, II, III, IV refer before and after fertilizer application as top-dressing at initiation of tiller and young panicle formation stage, respectively. (I) initiation of tiller stage, (II) 6 days after fertilizer application at initiation of tiller stage, (III) young panicle formation stage, (IV) 6 days after fertilizer application at young panicle formation stage. Seedling nitrogen treatment was started from sowing date on 13 June 2017 to 13 July 2017. L nitrogen indicates N supply level at 1/3 lower than that of the control during seedling stage.

Table S1. Primer sequences were used for quantitative real-time PCR in this study.

Gene name	Rap ID	Sequence	Annealing temperature (°C)
OsActin	Os03g0718100	F 5′-gactctggtgatggtgtcagc-3′<br>R 5′-ggctggaagaggacctcagg-3′	55.7
OsAMT1.1	LOC_Os04g43070	F 5′-tcatccctcaggtggtcatcg-3′<br>R 5′-cttggagatcttgggcagcag-3′	59.4
OsAMT1.2	LOC_Os02g40710	F 5′-ttcagcaggagaagccgtcagc-3′<br>R 5′-ccggcgtttatttgaggcaagc-3′	59.4
OsAMT1.3	LOC_Os02g40730	F 5′-aacaatcgaagcgcgagaaa-3′<br>R 5′-gcctgtagttggcacccaga-3′	69.5
OsAMT2.1	LOC_Os05g39240	F 5′-tagataggccttggaatcgcacc-3′<br>R 5′-tagagttcccattccaaaacaaacc-3′	57.3
OsAMT2.2	LOC_Os01g61510	F 5′-aacgtggctgctccttgaa-3′<br>R 5′-ttggcaataagccacacaca-3′	60.0
OsAMT2.3	LOC_Os01g61550	F 5′-atgctagagtttgaccgc-3′<br>R 5′-agtgtgatggatcctgcc-3′	65.3
OsAMT3.1	LOC_Os01g65000	F 5′-caccaagttcggcgacaagac-3′<br>R 5′-ctgcgtactacacgacgattgattc-3′	59.0
OsAMT3.2	LOC_Os03g62200	F 5′-aacaatcgaagcgcgagaaa-3′<br>R 5′-gcctgtagttggcacccaga-3′	54.9
OsAMT3.3	LOC_Os02g34580	F 5′-tagataggccttggaatcgcacc-3′<br>R 5′-tagagttcccattccaaaacaaacc-3′	50.9
OsNRT2.1	LOC_Os02g02170	F 5′-aacgtggctgctccttgaa-3′<br>R 5′-ttggcaataagccacacaca-3′	52.9
OsNRT2.1	LOC_Os02g02190	F 5′-atgctagagtttgaccgc-3′<br>R 5′-agtgtgatggatcctgcc-3′	52.9
OsNRT2.3a	LOC_Os01g50820	F 5′-caccaagttcggcgacaagac-3′<br>R 5′-ctgcgtactacacgacgattgattc-3′	59.0
OsNRT2.4	LOC_Os01g36720	F 5′-aacaatcgaagcgcgagaaa-3′<br>R 5′-gcctgtagttggcacccaga-3′	57.9
